# Notes and News

**Published:** 1896-09-12

**Authors:** 


### NOTES AND NEWS.

(Collected and Communicated.)

A severe epidemic of scarlet fever is reported in North Paddington.

Mr. J. G. Bryant, late of the Great Northern Central and Charing Cross Hospitals, has been appointed secretary of the Central London Ophthalmic Hospital, Gray's Inn Road.

Cholera in Egypt is now practically confined to Upper Egypt. There has been lately a temporary increase of cases in Cairo, but that is now abating, and the disappearance of the disease is now hopefully looked forward to.

Sir William Stokes has been elected president, and Mr. W. Thomson (president of the Royal College of Surgeons, Ireland) has been elected secretary and treasurer, of the Irish Committee of the International Medical Congress to be held in Moscow.

The Countess Cadogan paid a visit to the Royal Hospital for Incurables, Donnybrook, Dublin, last week. She was much pleased with her inspection and the general cheerfulness of the inmates. Amongst these is a survivor of the Crimean and of the China campaigns.

The Queen has granted Mr. Water Halliburton Macdonald, the chief medical officer of the British East Africa Protectorate, permission to wear [illegible] decoration of the Order of the Brilliant Star [illegible] the second class, conferred upon him by the late Sultan of Zanzibar in recognition of his services when employed in his service.

Mr. Newton H. Nixon, secretary of University College Hospital, writes to suggest that a large Volunteer Tournament should be organised yearly, on the lines of the Regular Army Tournament as held at Islington, for the benefit of the London hospitals, the proceeds to be distributed by the Metropolitan Hospital Sunday Fund. Mr. Nixon is himself a volunteer officer of long standing, and will be glad to hear that his suggestion is favourably entertained.

The new Consumptive Home for Scotland, near Glasgow, was formally opened by Lady Glen Coats last week. The new buildings have been erected free of debt at a cost of £17,000, and are only the commencement of a series intended to be added under Mr. Quarrier's scheme.

We are glad to see that *Truth* is protesting against a most unpleasant encroachment upon holiday-makers practised at an hotel at Harrogate. A guest is told off each Sunday to address the guests whilst at dinner on behalf of some charity. There appears to be no guarantee that the pleader knows anything about the management of the charity for which he pleads.

Professor Bouchard and M.M. Chauveau and d'Arsonval have been appointed on a Special Committee by the Academy of Science in Paris to report on the alleged cure for consumption practised by their countryman, Dr. Crotte. His method comprises the use of electricity and antiseptics. Dr. Crotte is reported to state that the electricity is employed to open the way for the microbe-destroying antiseptic.

We think the decision arrived at to abandon the proposed exhibition in aid of the Newcastle Infirmary is a wise one. A scheme which demanded a guaranteed sum of £10,000, the returns upon which are highly problematical, does not commend itself as financially prudent. The doubts which were in the minds of those most in favour of the project were shown by the fact that the suggestion arose that the guarantee should be regarded as a gift. Surely if £10,000 were forthcoming so easily it might be presented to the infirmary direct. Exhibitions have not often yielded £10,000 profit for the projectors.

We are informed that the committee of the Royal Free Hospital, Gray's Inn Road, have received through their secretary, Mr. Conrad W. Thies, a very liberal donation, £1,700, from Mr. John Bentley, late of Highbury Grange, to enable them to pay off the debt remaining on the new front buildings and other improvements which have recently been carried out at a total cost of over £30,000, and which were publicly opened by T.R.H. the Prince and Princess of Wales last year. The committee have decided to name one of the new wards "The John Bentley Ward" as a token of their appreciation of Mr. Bentley's liberal benefactions to the Royal Free Hospital.

A correspondent informs us that much dissatisfaction exists in medical circles in Edinburgh regarding the recent changes in the arrangements and editorship of the "Edinburgh Medical Journal." A large and influential meeting, representative of all branches of the profession in Edinburgh, was held this week to protest against the changes and the methods by which they have been brought about. It was decided *inter alia* to establish a new journal representative of the work of the Scottish centres of medical activity, and for that purpose a strong committee has been appointed. A substantial guarantee fund is already in existence, and the practical and financial support of the whole body of the profession has already been heartily given to the movement.

